# Methyl 3-carboxy-5-nitrobenzoate

**DOI:** 10.1107/S1600536809001093

**Published:** 2009-01-17

**Authors:** Pei Zou, Min-Hao Xie, Shi-Neng Luo, Ya-Ling Liu, Yong-Jia Shen

**Affiliations:** a Institute of Fine Chemicals, East China University of Science and Technology, Shanghai 200237, People’s Republic of China; b Jiangsu Institute of Nuclear Medicine, Wuxi 214063, People’s Republic of China

## Abstract

The structure of the title compound, C_9_H_7_NO_6_, is essentially planar [maximum deviation 0.284 (2)Å] except for the methyl H atoms. The crystal structure is stabilized by asymmetric O—H⋯O hydrogen bonds linking the hydrogen carboxyl­ates into pairs around the inversion centres. There is also π–π stacking of the benzene rings [centroid–centroid distance 3.6912 (12) Å].

## Related literature

The title complex is as an important inter­mediate for the preparation of iodinated X-ray contrast media, see: Morin *et al.* (1987 ▶); Singh & Rathore (1980 ▶); Stacul (2001 ▶); Jin & Xiao (2005 ▶).
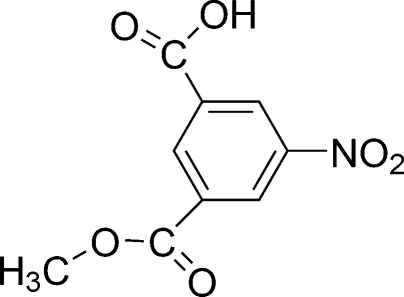


## Experimental

### 

#### Crystal data

C_9_H_7_NO_6_
*M* *_r_* = 225.16
Monoclinic, 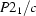

*a* = 7.3450 (15) Å
*b* = 8.9050 (18) Å
*c* = 14.474 (3) Å
β = 91.18 (3)°
*V* = 946.5 (3) Å^3^
*Z* = 4
Mo *K*α radiation
μ = 0.14 mm^−1^
*T* = 293 (2) K
0.30 × 0.20 × 0.10 mm

#### Data collection

Enraf–Nonius CAD-4 diffractometer
Absorption correction: ψ scan (North *et al.*, 1968 ▶) *T*_min_ = 0.950, *T*_max_ = 0.977
1859 measured reflections
1717 independent reflections
1284 reflections with *I* > 2σ(*I*)
*R*_int_ = 0.021
3 standard reflections every 200 reflections intensity decay: 1.0%

#### Refinement

*R*[*F* ^2^ > 2σ(*F* ^2^)] = 0.039
*wR*(*F* ^2^) = 0.105
*S* = 1.03
1717 reflections
150 parameters
H atoms treated by a mixture of independent and constrained refinement
Δρ_max_ = 0.17 e Å^−3^
Δρ_min_ = −0.13 e Å^−3^

### 

Data collection: *CAD-4 Software* (Enraf–Nonius, 1989 ▶); cell refinement: *CAD-4 Software*; data reduction: *XCAD4* (Harms & Wocadlo, 1995 ▶); program(s) used to solve structure: *SHELXS97* (Sheldrick, 2008 ▶); program(s) used to refine structure: *SHELXL97* (Sheldrick, 2008 ▶); molecular graphics: *SHELXTL* (Sheldrick, 2008 ▶); software used to prepare material for publication: *SHELXL97*.

## Supplementary Material

Crystal structure: contains datablocks I, global. DOI: 10.1107/S1600536809001093/fb2119sup1.cif

Structure factors: contains datablocks I. DOI: 10.1107/S1600536809001093/fb2119Isup2.hkl

Additional supplementary materials:  crystallographic information; 3D view; checkCIF report

## Figures and Tables

**Table 1:** Hydrogen-bond geometry (Å, °)

*D*—H⋯*A*	*D*—H	H⋯*A*	*D*⋯*A*	*D*—H⋯*A*
O6—H6*B*⋯O5^i^	0.95 (3)	1.67 (3)	2.6206 (19)	177.9 (17)
C8—H8*A*⋯O2^ii^	0.93	2.48	3.406 (2)	174

Symmetry codes: (i) 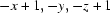; (ii) 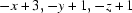.

**Fig. 1.**
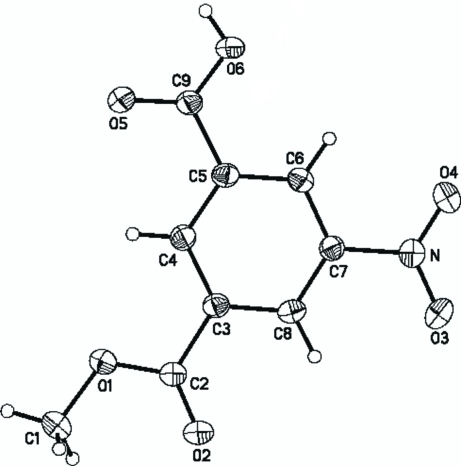
A view of the title compound with the atomic numbering scheme. Displacement ellipsoids were drawn at the 30% probability level.

**Fig. 2.**
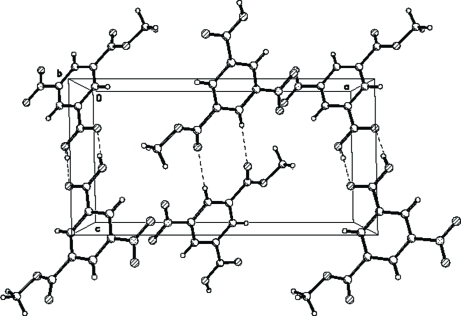
A packing diagram viewed along the b axis.

## supplementary crystallographic information

### Comment

The molecule of the title complex (Fig.1) is useful as an important intermediate for the preparation of iodinated X-ray contrast media, such as iotalamic acid, ioxitalamic acid, and Ioxilan, which are used clinically all over the world (Morin *et al.*, 1987; Singh *et al.*, 1980; Stacul *et al.*, 2001). We report here the crystal structure of title compound. The crystal data show that the bond lengths and angles are within expected ranges. TThe molecule is essentially planar: the maximum deviation from the weighted least-squares plane calculated through all the non-H atoms is 0.284 (2)Å for O2. The molecules are stacked *via*π-π interactions, with the centroid–centroid distance of 3.6912 (12)Å [symmetry code(i):  2-*x*, 1-*y*, 1-*z*]. The stacked columns are linked together by two intermolecular hydrogen bonds, O—H···O and C—H···O (Tab. 1 and Fig. 2). The O—H···O hydrogen bonds bind the hydrogencarboxylates into pairs.

### Experimental

Dimethyl 5-nitroisophthalic acid (956 mg, 4 mmol) was dissolved in hot methanol (6 ml), then sodium hydroxide (152 mg, 3.8 mmol) in methanol (2 ml) was added and refluxed for 30 min. Methanol was distilled off. The solid residue was extracted by warm water and the undissolved diester was filtered off. The filtrate was acidified with 1 mol/l hydrochloric acid (4 ml). The precipitate was filtered and washed with cold water. The crude product was purified by recrystallization. Single crystals were grown by slow evaporation of a ethanol/water (*v*/*v* 1:1) solution: colourless block-shaped crystals were formed after several days.

### Refinement

All the H atoms could have been discerned in the difference electron density maps. With exception of the hydrogen belonging to the hydroxyl group of the hydrogencarboxylate the hydrogens were situated into the idealized positions and refined in riding motion approximation. The hydroxyl hydrogen was refined freely. The used constraints: C_aryl_—H = 0.93 Å, *U*_iso_(H) = 1.2*U*_eq_(C_aryl_); C_methyl_—H  = 0.96 Å, *U*_iso_(H) = 1.5*U*_eq_(methyl).

### Crystal data

**Table:**

C_9_H_7_NO_6_	*F*(000) = 464
*M**_r_* = 225.16	*D*_x_ = 1.580 Mg m^−^^3^
Monoclinic, *P*2_1_/*c*	Mo *K*α radiation, λ = 0.71073 Å
Hall symbol: -P 2ybc	Cell parameters from 25 reflections
*a* = 7.3450 (15) Å	θ = 10–13°
*b* = 8.9050 (18) Å	µ = 0.14 mm^−^^1^
*c* = 14.474 (3) Å	*T* = 293 K
β = 91.18 (3)°	Block, colourless
*V* = 946.5 (3) Å^3^	0.30 × 0.20 × 0.10 mm
*Z* = 4	

### Data collection

**Table:**

Enraf–Nonius CAD-4 diffractometer	1284 reflections with *I* > 2σ(*I*)
Radiation source: fine-focus sealed tube	*R*_int_ = 0.021
graphite	θ_max_ = 25.3°, θ_min_ = 2.7°
ω/2θ scans	*h* = 0→8
Absorption correction: ψ scan (North *et al.*, 1968)	*k* = 0→10
*T*_min_ = 0.950, *T*_max_ = 0.977	*l* = −17→17
1859 measured reflections	3 standard reflections every 200 reflections
1717 independent reflections	intensity decay: 1.0%

### Refinement

**Table:**

Refinement on *F*^2^	Primary atom site location: structure-invariant direct methods
Least-squares matrix: full	Secondary atom site location: difference Fourier map
*R*[*F*^2^ > 2σ(*F*^2^)] = 0.039	Hydrogen site location: difference Fourier map
*wR*(*F*^2^) = 0.105	H atoms treated by a mixture of independent and constrained refinement
*S* = 1.03	*w* = 1/[σ^2^(*F*_o_^2^) + (0.057*P*)^2^ + 0.0354*P*] where *P* = (*F*_o_^2^ + 2*F*_c_^2^)/3
1717 reflections	(Δ/σ)_max_ < 0.001
150 parameters	Δρ_max_ = 0.17 e Å^−^^3^
0 restraints	Δρ_min_ = −0.13 e Å^−^^3^
23 constraints	

### Special details

**Table:**

Geometry. All e.s.d.'s (except the e.s.d. in the dihedral angle between two l.s. planes) are estimated using the full covariance matrix. The cell e.s.d.'s are taken into account individually in the estimation of e.s.d.'s in distances, angles and torsion angles; correlations between e.s.d.'s in cell parameters are only used when they are defined by crystal symmetry. An approximate (isotropic) treatment of cell e.s.d.'s is used for estimating e.s.d.'s involving l.s. planes.
Refinement. Refinement of *F*^2^ against ALL reflections. The weighted *R*-factor *wR* and goodness of fit *S* are based on *F*^2^, conventional *R*-factors *R* are based on *F*, with *F* set to zero for negative *F*^2^. The threshold expression of *F*^2^ > σ(*F*^2^) is used only for calculating *R*-factors(gt) *etc*. and is not relevant to the choice of reflections for refinement. *R*-factors based on *F*^2^ are statistically about twice as large as those based on *F*, and *R*- factors based on ALL data will be even larger.

### Fractional atomic coordinates and isotropic or equivalent isotropic displacement parameters (Å^2^)

**Table:**

	*x*	*y*	*z*	*U*_iso_*/*U*_eq_	
N	1.0387 (2)	0.49976 (17)	0.29780 (10)	0.0475 (4)	
O1	1.30752 (17)	0.20120 (16)	0.64959 (9)	0.0588 (4)	
C1	1.4451 (3)	0.2024 (3)	0.72271 (15)	0.0742 (7)	
H1A	1.4327	0.1142	0.7602	0.111*	
H1B	1.4301	0.2902	0.7603	0.111*	
H1C	1.5638	0.2036	0.6962	0.111*	
C2	1.3171 (2)	0.3110 (2)	0.58767 (12)	0.0442 (4)	
O2	1.4321 (2)	0.40512 (19)	0.58894 (10)	0.0769 (5)	
C3	1.1649 (2)	0.30531 (18)	0.51817 (11)	0.0375 (4)	
O3	1.1685 (2)	0.58281 (18)	0.29145 (11)	0.0730 (5)	
O4	0.9140 (2)	0.4969 (2)	0.24169 (11)	0.0832 (6)	
C4	1.0120 (2)	0.21563 (18)	0.53024 (11)	0.0382 (4)	
H4A	1.0053	0.1533	0.5816	0.046*	
O5	0.70372 (17)	0.03897 (15)	0.54893 (9)	0.0561 (4)	
C5	0.8694 (2)	0.21906 (18)	0.46585 (12)	0.0373 (4)	
O6	0.57474 (18)	0.13777 (16)	0.42237 (9)	0.0560 (4)	
H6B	0.475 (4)	0.072 (3)	0.4318 (19)	0.119 (10)*	
C6	0.8793 (2)	0.31082 (18)	0.38850 (11)	0.0380 (4)	
H6A	0.7850	0.3131	0.3447	0.046*	
C7	1.0324 (2)	0.39840 (18)	0.37824 (11)	0.0368 (4)	
C8	1.1757 (2)	0.39837 (18)	0.44108 (11)	0.0379 (4)	
H8A	1.2771	0.4589	0.4323	0.046*	
C9	0.7057 (2)	0.12519 (19)	0.48049 (12)	0.0396 (4)	

### Atomic displacement parameters (Å^2^)

**Table:**

	*U*^11^	*U*^22^	*U*^33^	*U*^12^	*U*^13^	*U*^23^
N	0.0515 (9)	0.0492 (9)	0.0419 (9)	−0.0049 (8)	−0.0003 (7)	0.0054 (7)
O1	0.0542 (8)	0.0634 (9)	0.0579 (8)	−0.0185 (7)	−0.0205 (6)	0.0167 (7)
C1	0.0644 (13)	0.1017 (19)	0.0555 (13)	−0.0228 (13)	−0.0249 (11)	0.0225 (13)
C2	0.0414 (9)	0.0467 (11)	0.0443 (10)	−0.0094 (9)	−0.0046 (8)	0.0030 (8)
O2	0.0626 (9)	0.0911 (12)	0.0759 (11)	−0.0421 (9)	−0.0269 (8)	0.0280 (9)
C3	0.0377 (9)	0.0344 (9)	0.0404 (9)	−0.0030 (7)	−0.0009 (7)	−0.0046 (7)
O3	0.0685 (10)	0.0765 (10)	0.0737 (10)	−0.0267 (8)	−0.0043 (8)	0.0321 (8)
O4	0.0783 (11)	0.1067 (14)	0.0635 (10)	−0.0288 (10)	−0.0274 (8)	0.0353 (9)
C4	0.0408 (9)	0.0331 (9)	0.0407 (9)	−0.0040 (7)	−0.0017 (7)	−0.0005 (7)
O5	0.0499 (8)	0.0578 (8)	0.0601 (8)	−0.0174 (6)	−0.0112 (6)	0.0174 (7)
C5	0.0369 (8)	0.0324 (9)	0.0424 (9)	−0.0027 (7)	−0.0020 (7)	−0.0053 (7)
O6	0.0454 (8)	0.0569 (9)	0.0650 (9)	−0.0201 (7)	−0.0184 (7)	0.0123 (7)
C6	0.0396 (9)	0.0369 (9)	0.0373 (9)	−0.0014 (8)	−0.0049 (7)	−0.0040 (7)
C7	0.0409 (9)	0.0355 (9)	0.0342 (8)	−0.0027 (7)	0.0020 (7)	−0.0005 (7)
C8	0.0332 (8)	0.0370 (9)	0.0436 (10)	−0.0037 (7)	0.0034 (7)	−0.0050 (8)
C9	0.0420 (9)	0.0330 (9)	0.0435 (10)	−0.0055 (8)	−0.0060 (8)	−0.0006 (8)

### Geometric parameters (Å, °)

**Table:**

N—O3	1.211 (2)	C4—C5	1.388 (2)
N—O4	1.2122 (19)	C4—H4A	0.9300
N—C7	1.475 (2)	O5—C9	1.254 (2)
O1—C2	1.329 (2)	C5—C6	1.389 (2)
O1—C1	1.448 (2)	C5—C9	1.483 (2)
C1—H1A	0.9600	O6—C9	1.270 (2)
C1—H1B	0.9600	O6—H6B	0.95 (3)
C1—H1C	0.9600	C6—C7	1.379 (2)
C2—O2	1.190 (2)	C6—H6A	0.9300
C2—C3	1.489 (2)	C7—C8	1.377 (2)
C3—C4	1.393 (2)	C8—H8A	0.9300
C3—C8	1.393 (2)		
			
O3—N—O4	123.18 (16)	C3—C4—H4A	119.9
O3—N—C7	118.11 (15)	C4—C5—C6	120.19 (15)
O4—N—C7	118.71 (15)	C4—C5—C9	119.62 (15)
C2—O1—C1	116.20 (15)	C6—C5—C9	120.19 (15)
O1—C1—H1A	109.5	C9—O6—H6B	115.3 (17)
O1—C1—H1B	109.5	C7—C6—C5	118.42 (15)
H1A—C1—H1B	109.5	C7—C6—H6A	120.8
O1—C1—H1C	109.5	C5—C6—H6A	120.8
H1A—C1—H1C	109.5	C8—C7—C6	122.86 (15)
H1B—C1—H1C	109.5	C8—C7—N	119.05 (15)
O2—C2—O1	123.71 (17)	C6—C7—N	118.05 (15)
O2—C2—C3	123.86 (17)	C7—C8—C3	118.28 (15)
O1—C2—C3	112.41 (15)	C7—C8—H8A	120.9
C4—C3—C8	120.06 (15)	C3—C8—H8A	120.9
C4—C3—C2	122.07 (15)	O5—C9—O6	123.82 (16)
C8—C3—C2	117.81 (15)	O5—C9—C5	118.73 (15)
C5—C4—C3	120.18 (16)	O6—C9—C5	117.45 (15)
C5—C4—H4A	119.9		
			
C1—O1—C2—O2	1.4 (3)	C5—C6—C7—N	−177.71 (14)
C1—O1—C2—C3	−177.00 (17)	O3—N—C7—C8	−2.1 (2)
O2—C2—C3—C4	−165.32 (18)	O4—N—C7—C8	178.43 (17)
O1—C2—C3—C4	13.1 (2)	O3—N—C7—C6	175.95 (17)
O2—C2—C3—C8	12.0 (3)	O4—N—C7—C6	−3.6 (2)
O1—C2—C3—C8	−169.58 (15)	C6—C7—C8—C3	0.1 (2)
C8—C3—C4—C5	−0.3 (2)	N—C7—C8—C3	178.04 (14)
C2—C3—C4—C5	176.95 (16)	C4—C3—C8—C7	−0.1 (2)
C3—C4—C5—C6	0.7 (2)	C2—C3—C8—C7	−177.46 (15)
C3—C4—C5—C9	−178.81 (15)	C4—C5—C9—O5	−3.4 (2)
C4—C5—C6—C7	−0.6 (2)	C6—C5—C9—O5	177.14 (16)
C9—C5—C6—C7	178.84 (15)	C4—C5—C9—O6	176.41 (16)
C5—C6—C7—C8	0.2 (2)	C6—C5—C9—O6	−3.1 (2)

### Hydrogen-bond geometry (Å, °)

**Table:**

*D*—H···*A*	*D*—H	H···*A*	*D*···*A*	*D*—H···*A*
O6—H6B···O5^i^	0.95 (3)	1.67 (3)	2.6206 (19)	177.9 (17)
C8—H8A···O2^ii^	0.93	2.48	3.406 (2)	174

Symmetry codes: (i) −*x*+1, −*y*, −*z*+1; (ii) −*x*+3, −*y*+1, −*z*+1.
